# Should I and Can I Use AI for Forensic Psychiatry Report Writing?

**DOI:** 10.2196/99749

**Published:** 2026-08-07

**Authors:** Alexandre Hudon

**Affiliations:** 1 Department of Psychiatry and Addictology, Faculty of Medicine, Université de Montréal, 2900 boulevard Edouard-Montpetit, Montreal, QC, H3T 1J4, Canada, 1 514-251-4000; 2 Centre de recherche de l'Institut universitaire en santé mentale de Montréal, Montreal, QC, Canada; 3 Department of Psychiatry, Institut national de psychiatrie légale Philippe-Pinel, Montreal, QC, Canada

**Keywords:** forensic psychiatry, large language models, psychology, medical documentation, expert witness, clinical decision support, mental health, AI ethics, health technology

## Abstract

The rapid evolution of AI, particularly large language models (LLMs), has renewed interest in their potential role in forensic psychiatry report writing. Recent evidence demonstrates that contemporary LLMs perform well in selected medical knowledge, documentation, and information management tasks and may reduce the administrative burden when deployed under appropriate clinical supervision. However, forensic psychiatric reports differ fundamentally from routine clinical documentation. They constitute expert evidence prepared for legal proceedings and therefore require transparent reasoning, explicit weighing of competing evidence, a robust factual foundation, and personal professional accountability. This viewpoint examines whether AI can and should be used in forensic psychiatry report writing by integrating recent empirical evidence, forensic psychiatry guidance, legal and regulatory frameworks, and emerging governance recommendations. Rather than comparing AI with an idealized human evaluator, the manuscript argues that the appropriate comparison is between 2 imperfect systems of reasoning. Human experts remain susceptible to cognitive biases, omission errors, and disagreement, whereas contemporary LLMs exhibit distinct vulnerabilities, including hallucinations, hidden omissions, probabilistic reasoning, and limited explainability. Although the mechanisms differ, both may ultimately compromise the reliability of expert evidence if left unchecked. Current evidence supports AI for bounded, reversible, and independently verifiable tasks, such as document organization, chronology construction, indexing, transcription, and structured summarization, particularly within secure and validated environments. By contrast, there remains insufficient evidence to support AI-assisted generation or material shaping of psycholegal reasoning, credibility assessments, or final forensic opinions. Because these activities require interpretation, accountability, and reasoning that can withstand judicial scrutiny, they remain fundamentally human responsibilities. The most defensible implementation model is, therefore, one of AI around the report rather than AI writing the report, in which AI serves as a supervised productivity tool while the forensic psychiatrist retains full authorship, accountability, and justification of all substantive conclusions.

## Why Are Forensic Psychiatry Reports Different?

Classical forensic writing literature and current forensic psychiatry guidance agree that a report is not a neutral record of facts. Rather, it is a disciplined professional opinion prepared for a legal audience. Older forensic report-writing literature emphasized comprehensiveness, scientific impartiality, and the need to avoid recurring failures such as unsupported conclusions, role confusion, and weak linkage between facts and legal questions. Contemporary forensic psychiatry guidance adds that the strength of a report depends on its factual foundation, the explicitness of its reasoning, and the expert’s ability to distinguish verified from unverified information and facts from inferences and impressions [1].

This is why the core forensic obligations are unusually resistant to automation. The ethics guidance of the American Academy of Psychiatry and the Law (AAPL) states that forensic psychiatrists should adhere to honesty, strive for objectivity, base reports on all available data, and distinguish verified from unverified information. The report-writing guidance of the Canadian Academy of Psychiatry and the Law similarly requires an objective and nonpartisan assessment based on all relevant information, the documentation of omissions, the disclosure of limitations, and an explicit nexus between the report’s data and its final opinions. Those requirements map poorly onto a technology whose primary competence is the fluent prediction of plausible language rather than accountable evidential reasoning [2].

Forensic practice also carries an established vulnerability to cognitive bias. A 2025 scoping review of forensic psychiatry identified 10 distinct cognitive biases across 24 studies, with gender bias, allegiance bias, and confirmation bias among the most frequently discussed, and concluded that structured methods are more promising for mitigation than simple self-awareness [3]. This finding is important because one of the appealing myths of AI is that it will neutralize human bias. In reality, AI does not eliminate bias. Rather, it shifts important components of bias from individual clinicians’ cognition to training data, model architecture, reinforcement procedures, interface design, retrieval pipelines, and deployment context.

The upshot is that forensic report writing is a poor candidate for “set-it-and-forget-it” automation. It is a better candidate for carefully controlled support around the edges of the reasoning process. A useful perspective is that any application that makes the report more explicit, traceable, and easier to check may be compatible with forensic standards, whereas any application that makes it harder to know why a conclusion was reached is presumptively suspect [4]. This perspective is presented in Table 1.

**Table 1.** Key elements of forensic psychiatric reports and AI implications.

Forensic requirement	What the report must demonstrate	Why this matters for AI use
Objectivity	The expert’s view must not be distorted by the retaining party and must distinguish verified from unverified information [2].	A model that drafts analysis can silently import framing, bias, or omitted caveats.
Adequate factual foundation	Opinions are only as strong as the facts on which they rest, and omissions must be documented [4].	Current ambient documentation studies consistently identify omission errors among the most frequent AI-generated documentation errors, although omission errors are also well recognized in human clinical documentation. The distinguishing concern is not simply the occurrence of omissions but their potentially systematic invisibility because fluent AI-generated narratives may conceal missing evidence unless outputs are explicitly verified against source material.
Explicit reasoning	The report should show a logical path from data to psycholegal conclusion [4].	Opaque AI assistance can weaken the visible chain of reasoning and invite challenge.
Disclosure of limits	Lack of personal examination, missing collateral, unreliable participation, and other limits must be clearly stated [2].	AI outputs often understate uncertainty unless the workflow forces explicit qualification.
Confidentiality and notice	Forensic evaluees and collateral sources require notice about purpose and limits of confidentiality, with consent where necessary and feasible [2].	Uploading identifiable material to public AI tools can defeat these obligations.

Throughout this manuscript, “consumer cloud-based LLMs” refers to publicly accessible internet-hosted generative AI services in which prompts and uploaded content are processed on external vendor infrastructure. This definition is independent of whether the service is free or subscription-based.

## What AI Can Do Now

Technically, the case for limited AI use is increasingly supported by empirical evidence, although that evidence remains highly task-dependent. Early benchmark studies demonstrated that contemporary large language models (LLMs) perform strongly on medical knowledge and clinical reasoning tasks. In a 2024 evaluation of GPT-3.5 and GPT-4 against official medical board examinations, GPT-4 achieved its highest relative performance in psychiatry, scoring at approximately the 75th percentile among physicians [5]. Similarly, GPT-4 demonstrated higher diagnostic accuracy than resident physicians in simulated emergency department cases [6]. However, these studies primarily compared LLMs with trainees or early-career clinicians rather than experienced specialists, and none evaluated performance against board-certified forensic psychiatrists performing psycholegal assessments.

Evidence has subsequently expanded beyond examination performance to real-world clinical implementation. Recent reviews conclude that LLMs show considerable promise for administrative support, education, documentation, clinical decision support, and information retrieval within psychiatry, while emphasizing that the evidence remains heterogeneous and insufficient to support autonomous clinical practice [7]. Likewise, evaluations of commercial AI documentation tools for mental health report substantial variability in functionality, transparency, privacy safeguards, and governance, with relatively few products performing well across technical, security, and ethical domains [8]. Collectively, the literature suggests that current AI systems are best viewed as productivity tools rather than independent clinical decision-makers.

These strengths are particularly relevant to the preparatory stages of forensic report writing. LLMs can efficiently organize large case files, identify relevant information, classify documents, summarize lengthy records, extract structured variables, and generate chronologies. A recent forensic psychiatry case study using GPT-4o demonstrated the feasibility of extracting both clinical and nonclinical variables from forensic expert reports, supporting the model’s potential role in dossier preparation and structured information management while stopping short of recommending automated medicolegal opinion generation [9]. Consistent with this distinction, the Academy of Experts considers document organization, transcription, indexing, and summarization of nonsensitive materials to represent substantially lower-risk applications than drafting expert opinions or providing legal conclusions [10].

The strongest empirical evidence currently comes from AI-assisted clinical documentation. A large multicenter quality-improvement study evaluating ambient AI scribes across 6 health systems found significant reductions in clinician burnout, cognitive workload, and after-hours documentation following implementation [11]. Similar implementation studies published in 2026 also demonstrated meaningful efficiency gains, although clinically important documentation errors remained [12,13]. Importantly, studies specifically evaluating documentation quality consistently report that current LLMs continue to generate hallucinations, omit clinically relevant information, and occasionally produce serious errors despite improvements in newer models. For example, GPT-4 generated hallucinations in approximately 42% of clinical summaries and omitted at least one clinically relevant item in 47% of summaries, whereas GPT-3.5 exhibited even higher hallucination rates (64%) [14]. Even GPT-4 Turbo, despite producing fewer hallucinations than physicians, generated approximately twice as many omissions as human clinicians when composing discharge narratives [15]. More recent evaluations of GPT-4, Gemini 2.5 Pro, and other contemporary models demonstrate declining hallucination rates but persistent omission errors, indicating that improvements in factual accuracy have not eliminated clinically meaningful failures [16-18]. The practical implications of these studies for forensic psychiatry are summarized in Table 2.

However, a forensic psychiatric report differs fundamentally from a routine clinical note. Clinical documentation primarily records observations and management, whereas forensic reports require interpretation of conflicting evidence, assessment of credibility, explicit consideration of alternative explanations, application of legal standards, transparent management of uncertainty, and reasoning that can withstand adversarial scrutiny. Errors in this context extend beyond factual inaccuracies to include inappropriate weighting of evidence, omission of legally relevant information, flawed causal reasoning, and unsupported psycholegal conclusions. Consequently, although current evidence supports the use of AI for reversible administrative tasks such as chronology construction, indexing, transcription, and summarization, direct evidence supporting AI-assisted psycholegal reasoning remains extremely limited. Moving from administrative assistance to substantive authorship of forensic opinions would therefore extend well beyond the available empirical evidence and should currently be considered unsupported.

**Table 2.** Overview of the main findings and example practical implications for forensic report writing.

Study	Model	Design and domain	Main finding	Hallucinations	Omissions	Serious errors	Human comparator	Practical meaning for forensic report writing
Katz et al [5], 2024, NEJM AI	GPT-3.5; GPT-4	Comparative evaluation of LLMs^a^ on medical knowledge and reasoning	GPT-4 substantially outperformed GPT-3.5 and approached physician-level performance on several reasoning tasks.	Not assessed	Not assessed	Not assessed	Resident physicians; board examination scores	Useful only as a general reasoning benchmark; does not validate forensic report writing.
Hoppe et al [6], 2024, JMIR	GPT-3.5; GPT-4	Diagnostic accuracy study in emergency medicine	GPT-4 demonstrated higher diagnostic accuracy than GPT-3.5 but remained susceptible to clinically relevant reasoning errors.	Not assessed	Not assessed	Diagnostic errors reported through accuracy metrics	Emergency physicians	Demonstrates diagnostic reasoning potential, but forensic opinions require source-grounded evidentiary analysis.
Petroni et al [9], 2025, Int J Law Psychiatry	GPT-4o	Forensic psychiatry case study	GPT-4o successfully summarized and organized forensic material but required expert oversight for interpretation.	Not reported	Not reported	Not reported	None	Directly relevant to forensic psychiatry; supports information extraction and summarization rather than autonomous expert opinion.
Olson et al [11], 2025, JAMA Netw Open	Ambient AI scribe; underlying model not specified	Clinical implementation study of ambient AI documentation	Ambient AI substantially reduced documentation burden while maintaining acceptable documentation quality.	Not quantified	Not quantified	Not quantified	Pre-post clinician outcomes	Supports administrative efficiency but does not address forensic accuracy or legal-risk questions.
Bouguettaya et al [8], 2025, Gen Hosp Psychiatry	Review of AI note tools; models often undisclosed	Narrative review of AI-assisted clinical documentation	Documentation quality generally improved, although governance, transparency, and error reporting varied considerably.	Reviewed as risk; not pooled	Reviewed as risk; not pooled	Reviewed as risk; not pooled	Not applicable	Highlights that undisclosed models and heterogeneous evaluation methods are incompatible with forensic transparency requirements.
Taylor et al [12], 2026, JMIR Medical Informatics	Ambient listening generative AI; underlying model not specified	Multicenter evaluation of ambient AI clinical notes	Most AI-generated notes were acceptable, although clinically important errors remained uncommon but present.	Not separately reported	Not separately reported	19/356 notes (5.3%) contained risk-of-harm errors	No concurrent human comparator	Even supervised AI notes contain clinically meaningful errors; forensic reports require complete verification.
Reddy et al [13], 2026, Annals Preview	Ambient AI tools; model/vendor not specified in preview	Standardized clinical documentation evaluation	AI-generated notes scored consistently below physician-generated notes across standardized scenarios.	Not separately reported	Not separately reported	AI notes scored lower than human notes across 5 standardized cases	Human-generated notes	Suggests that current ambient AI documentation remains insufficient for high-stakes forensic reporting without expert revision.
Hua et al [7], 2025, NPJ Digital Medicine	Multiple LLMs	Systematic review of LLMs in mental health	Current evidence supports potential clinical utility but emphasizes limited validation, heterogeneous methods, and persistent safety concerns.	Discussed; not pooled	Discussed; not pooled	Discussed; not pooled	Review-level evidence	Indicates that evidence supporting LLM use in psychiatry remains preliminary and insufficient for independent forensic use.
Hartman et al [19], 2024, JAMA Network Open	Llama-2 7B	Evaluation of AI-generated clinical handoff summaries	AI-generated summaries demonstrated good safety but lower completeness than human documentation.	Not quantified; safety score high	Not quantified; completeness lower	No critical safety risks	Physician handoff notes	Supports AI for structured administrative summaries, but not for independent forensic opinions.
Williams et al [14], 2025, PLOS Digital Health	GPT-4‐0613	Controlled evaluation of AI-generated clinical documentation	GPT-4 reduced hallucinations compared with GPT-3.5 but omissions remained frequent.	42% with ≥1 hallucination	47% with ≥1 omission	3 potentially harmful errors	No concurrent human comparator	Even strong models omit legally relevant facts; forensic use requires line-by-line source verification.
Williams et al [14], 2025, PLOS Digital Health	GPT-3.5-turbo-0613	Controlled evaluation of AI-generated clinical documentation	GPT-3.5 showed substantially higher hallucination rates than GPT-4.	64% with ≥1 hallucination	50% with ≥1 omission	Not separately reported	No concurrent human comparator	Older models are too error-prone for forensic report drafting beyond low-risk administrative support.
Williams et al [15], 2025, JAMA Internal Medicine	GPT-4-turbo-128K	Comparative study of discharge narrative generation	GPT-4 produced fewer hallucinations than physicians but significantly more omissions.	0.23 per narrative vs 0.31 for physicians	1.75 per narrative vs 0.86 for physicians	6 serious LLM errors; 1 harmful narrative	Physician discharge summaries	The principal forensic concern is omission rather than hallucination because missing collateral information may alter legal conclusions.
Asgari et al [16], 2025, NPJ Digital Medicine	GPT-4‐32k-0613	Prospective evaluation of structured AI documentation workflow	Workflow engineering substantially reduced documentation errors.	1.47% overall	3.45% overall	Reduced through workflow engineering	Prior human benchmark only	Suggests that structured workflows can improve safety, but forensic reports still require auditable human review.
Song et al [17], 2025, JAMA Network Open	Llama3-8B-based model	Comparative evaluation of AI-assisted clinical note generation	AI assistance reduced documentation workload while introducing occasional factual confabulations.	1 confabulation/50 notes	6 omissions/50 notes	One confabulation described procedures that had not occurred	Manual notes and LLM-assisted notes	AI-assisted drafting may improve efficiency, but fabricated facts remain unacceptable in forensic reports.
Grolleau et al [18], 2026, JAMA Network Open	Gemini 2.5 Pro	Evaluation of AI-generated clinical summaries	Hallucinations were uncommon, whereas omissions remained frequent.	2/100 summaries	25/100 summaries	1 moderate-harm summary	Physician review only	Even state-of-the-art models continue to omit clinically relevant information; newer models should not be presumed forensic-grade.
Lieslehto et al [20], 2026, Scientific Reports	Sentence-BERT^c^+ SVM^d^	Machine learning analysis of forensic psychiatric reasoning	NLP^b^ methods successfully characterized reasoning patterns within forensic assessments.	Not applicable	Not applicable	Not applicable	None	Useful for studying forensic reasoning and quality assurance, but not for drafting individual forensic reports.

a LLM: large language model.

b NLP: natural language processing.

c BERT: Bidirectional Encoder Representations from Transformers.

d SVM: support vector machine.

## Why Is the Evidence Not Enough for Autonomous Report Writing?

The principal limitation of current generative AI systems is not linguistic fluency but epistemic reliability. Contemporary large language models are optimized to generate statistically plausible continuations of text rather than to establish whether individual statements are supported by sufficient evidence. Consequently, outputs may appear coherent, internally consistent, and professionally written while containing unsupported inferences, omitted evidence, incorrect citations, or fabricated information. The National Institute of Standards and Technology (NIST) explicitly identifies confabulation (hallucination) as a core risk of generative AI and recommends independent verification of outputs, provenance tracking, and human review as essential safeguards rather than optional quality-improvement measures. This distinction is particularly important in forensic psychiatry, where the legal value of an opinion depends less on rhetorical coherence than on the transparency of the evidentiary chain linking observed facts to psycholegal conclusions [21]. These are not minor implementation details. In forensic psychiatry, they are the difference between defensible assistance and an opinion that cannot survive disclosure or cross-examination.

Omission is arguably the most dangerous documentation error for forensic work because it can leave the report coherent, fluent, and apparently balanced while silently removing a disconfirming fact. In the 2026 AI-note pilot, omissions were the most frequent error category, and hallucinations were also common [12]. A small proportion of notes contained errors deemed to pose serious or imminent risk if left uncorrected. Controlled evaluations in primary care likewise found AI notes inferior to human notes. The forensic implication is severe: an omitted sentence about collateral inconsistency, medication nonadherence, past malingering, intoxication, or contradictory witness material can alter the meaning of the whole report.

Bias is the second major limitation. A 2024 Lancet Digital Health evaluation repurposed clinical cases to test demographic effects and found that changing race or ethnicity, as well as gender, affected GPT-4 outputs in clinical decision scenarios [22]. A forensic psychiatry bias review identified allegiance, confirmation, gender, hindsight, cultural, and emotional biases as persistent concerns in human evaluation. In criminal justice more broadly, the literature on algorithmic recidivism prediction offers a cautionary precedent: one influential 2018 study argued that the widely used COMPAS (Correctional Offender Management Profiling for Alternative Sanctions) tool was no more accurate or fair than predictions made by people with little or no expertise [23]. The lesson is not that humans are unbiased and AI is biased. The lesson is that bias remains a design, governance, and methodology problem whether the agent is human or algorithmic.

A further problem is narrative style. Forensic reports do persuasive work through tone, qualification, sequencing, and emphasis [24]. Because older forensic scholarship treats reports as narrative instruments that must be carefully calibrated for a legal audience, stylistic interference is not trivial. Even apparently harmless assistance with wording can subtly change how certainty, credibility, empathy, blame, or danger are conveyed. That is precisely why expert-witness guidance now treats AI-generated narrative content as high risk and AI-generated substantive analysis as an extreme risk or prohibited.

**Figure 1.**
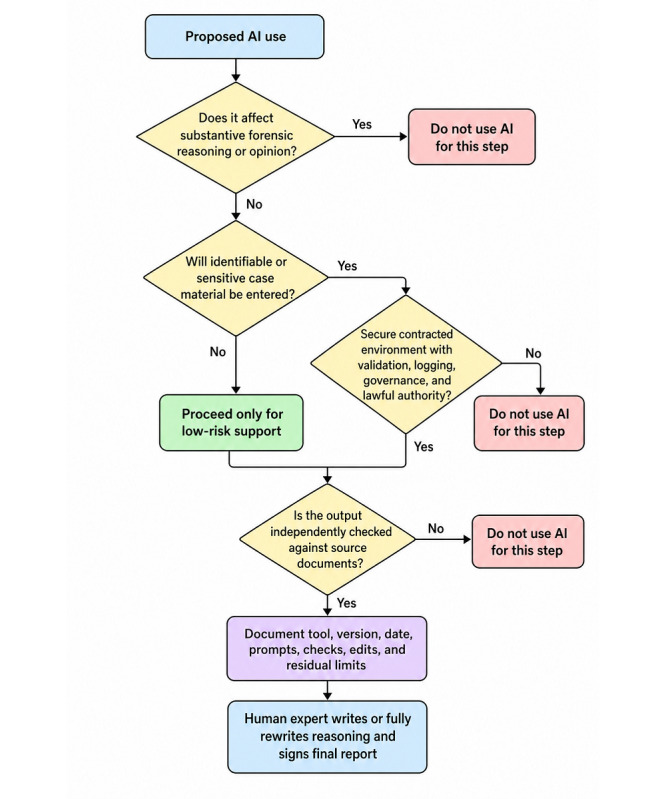
Decision rule for the use of AI in forensic report writing.

The decision rule shown in Figure 1 reflects the convergent logic of forensic ethics, expert witness guidance, privacy guidance, and generative AI risk management: bounded support may be acceptable, but a hidden or uncheckable contribution to expert reasoning is not.

## Legal, Ethical, Confidentiality, and Professional Responsibility Issues

Across medicine and mental health, official guidance is increasingly aligned on human accountability. The position statement of the American Psychiatric Association recognizes potential uses of AI, including assistance with clinical documentation, while its educational guidance emphasizes legal responsibility, transparency, and informed decision-making [25]. The 2025 ethical guidance of the American Psychological Association likewise frames AI as a practice issue requiring real-world safeguards. At the global level, the World Health Organization has articulated 6 core AI-for-health principles and, in its guidance on large multimodal models, has called for broad recommendations to ensure appropriate and safe use of AI in health care [26].

For forensic psychiatrists, confidentiality and role clarity create an even sharper boundary. AAPL ethics require notifying the evaluee and collateral sources regarding the purpose of the evaluation and the limits of confidentiality, and obtaining informed consent when necessary and feasible [2]. This means that inserting a third-party generative model into the workflow is not just a technical procurement choice. It may alter who receives the information, whether confidentiality is preserved, whether consent or lawful authority is adequate, and whether the evaluator has created a discoverable trail outside the expected forensic channel.

Recent court and expert guidance is explicit about these risks. Guidance from the Federal Court requires the declaration of AI-generated content in litigation materials and states that expert reports should disclose AI use in the methodology summary; it also stresses the hallucination risk, verification, and the signer’s responsibility for accuracy [27]. Guidance from the Canadian Judicial Council states that no judge may delegate decision-making authority to a computer program, requires explainability, a formal impact assessment, security controls, and ongoing monitoring, and warns against uploading sensitive material to free AI sites. The 2026 guidance from the Academy of Experts classifies AI-generated substantive analysis or opinion as an extreme risk, warns that the use of public AI can breach confidentiality and privilege, and states that writing the entire expert report through AI is a clear violation of expert duties [28]. Guidance from the Judiciary of England and Wales emphasizes that consumer cloud-based LLMs are a poor way to find new information that cannot be independently verified and warns that inputs to consumer cloud-based LLMs should be treated as if they were published to the world [29].

Health sector privacy and nondiscrimination guidance points in the same direction. The Information and Privacy Commissioner of Ontario advises contractual safeguards, accountability structures, vendor assessment, and ongoing monitoring for AI scribe use. The College of Physicians and Surgeons of Ontario states that physicians must inform patients about AI use, obtain consent before recording conversations, and remain ultimately accountable for decision support and documentation [30]. The College of Physicians and Surgeons of British Columbia similarly does not endorse specific AI tools and notes that evidence remains limited [31]. In the United States, the US Department of Health and Human Services (HHS) Office for Civil Rights (OCR) states that Section 1557 requires covered health programs that use AI in patient care to take reasonable steps to identify and mitigate discrimination risk, while proposed HIPAA (Health Insurance Portability and Accountability Act) Security Rule changes emphasize conducting a written risk analysis when adopting new technology assets that may affect electronic protected health information [32].

The regulatory context also means that not every AI tool is regulated in the same way. Some note-support or nondevice clinical decision support functions may sit outside classic medical device regulation, while AI products that diagnose, treat, or function as digital mental health medical devices may fall within Food and Drug Administration–style oversight. For forensic psychiatrists, this creates a practical point: the absence of device regulation does not imply safety, validity, or fitness for forensic use [33]. It often means the validation burden shifts back onto the deploying organization and, ultimately, onto the professional using the system. All the guidelines and their takeaway messages for forensic psychiatry report writing are summarized in Table 3.

**Table 3.** Guidelines and core messages for forensic psychiatric writing.

Guideline or regulatory source	Core message	Takeaway for forensic psychiatry report writing
AAPL^a^ ethics [2]	Honesty, objectivity, distinction between verified and unverified information, notice of purpose and limits of confidentiality	AI cannot write what the expert must personally own and justify
CAPL^b^ report-writing guidance [4]	Objective and nonpartisan assessment, explicit reasoning, factual foundation, disclosure of limitations	AI may assist workflow, but the report’s reasoning chain must remain transparent and human
WHO^c^ AI-for-health guidance [26]	Autonomy, safety, transparency, accountability, equity, sustainability	“Should I” is an ethics-and-governance question, not just a productivity question
APA^d^ psychiatry and psychology guidance [25]	AI can support care and administration, but clinicians remain responsible	Mental health professionals must supervise, explain, and verify
Federal Court of Canada notice [27]	AI-generated litigation content may require declaration; expert reports should disclose AI in methodology; signer remains responsible	Material AI participation in a report may need explicit disclosure
Canadian Judicial Council guidance [27]	No delegation of judicial decision-making, need explainability, security, and impact assessment	By analogy, forensic experts should avoid delegating expert reasoning to opaque tools
Academy of Experts guidance [28]	Opinion drafting is extreme risk; whole-report drafting is prohibited; confidentiality and privilege are at stake	The expert’s opinion must remain authentically the expert’s own
UK judiciary guidance [29]	Consumer cloud-based LLMs^e^ are poor for unverifiable research and unsafe for confidential information	Public AI tools are unsuitable for identifiable forensic case material
Ontario IPC^f^ and CPSO^g^ guidance [30]	Governance, contracts, consent, clinician accountability	Safe deployment requires institutional controls, not individual improvisation
HHS^h^ OCR^i^ and HIPAA^j^ materials [32]	AI use can create discrimination and security obligations	Bias audits and privacy risk assessments are not optional in regulated environments

a AAPL: American Academy of Psychiatry and the Law.

b CAPL: Canadian Academy of Psychiatry and the Law.

c WHO: World Health Organization.

d APA: American Psychiatric Association.

e LLM: large language model.

f IPC: Information and Privacy Commissioner of Ontario.

g CPSO: College of Physicians and Surgeons of Ontario.

h HHS: US Department of Health and Human Services.

i OCR: Office for Civil Rights.

j HIPAA: Health Insurance Portability and Accountability Act.

## A Safe Implementation Model for Clinicians

The most defensible implementation strategy is to separate low-risk clerical augmentation from high-risk epistemic work. AI may be reasonable for document indexing, chronology creation, deidentification support, record summarization into source-linked notes, extraction of dates and diagnoses from already reviewed files, or language polishing after the expert has already formed the opinion. It becomes much harder to justify when it proposes causation, psycholegal formulation, credibility judgments, malingering inferences, risk conclusions, or ultimate opinions. The Academy of Experts is right to treat substantive opinion drafting as an extreme risk, and current forensic ethics makes the same judgment by another route: the opinion must be honestly yours [28].

A second principle is that environment matters. Public consumer chatbots are the wrong default for forensic work because courts and regulators repeatedly warn that private or confidential information entered into them may be disclosed, reused, or lose privilege. A safer architecture is a contracted enterprise or API deployment with documented security controls, role-based access, logging, retention limits, and, where relevant, an equivalent contractual framework. Even then, compliance is never automatic. Health sector privacy guidance also stresses governance, provenance, measurement, and monitoring rather than blind trust in vendor labels [29].

A third principle is validation in the local use case. Before any AI-supported step is allowed near a live forensic report, the organization should run a local validation against historical cases or standardized mock files, measure omissions, hallucinations, bias, and time saved, and define acceptable and unacceptable failure thresholds. The Canadian Judicial Council recommends a pilot or sandbox approach for court deployment, and the AI scribe literature increasingly emphasizes exactly this kind of predeployment testing and ongoing auditing. In forensic psychiatry, a sensible test set would include cases with conflicting collateral, culturally complex presentations, malingering concerns, intoxication, poor records, and legally sensitive wording so that narrative risk is tested, not just clerical efficiency [34].

A fourth principle is documentation. If AI is used at all, the expert or institution should preserve the model or product name, version, date, task, scope, prompt class or template, the source documents used, the human reviewer, what was accepted or rejected, and what limitations remain. NIST explicitly recommends provenance tracking and review of citations and sources, while the Academy of Experts recommends contemporaneous records of why AI was used, what risks and safeguards were identified, and how outputs were handled [29,35]. This is not bureaucratic overkill. It is what turns AI use from invisible ghostwriting into auditable assistance.

These principles are a translation of the current literature into practice and are expanded upon in Table 4. Implementation steps are presented in Table 5.

**Table 4.** Tool classes and regulatory principles with their strengths and risks in forensic work.

Tool class	Typical strength	Main risk in forensic work	Relative suitability
Consumer cloud-based LLMs^a^	Fast drafting, quick background explanation	Confidentiality loss, unverifiable citations, unstable reasoning, no case-specific governance	Generally unsuitable for identifiable or sensitive forensic material
Enterprise cloud LLM with contract and audit features	Controlled access, logging, possible health data contractual support	Compliance depends on configuration, validation, and human oversight; still prone to omission and confabulation	Potentially suitable for low-risk support tasks if locally validated
Ambient documentation system	Can reduce clerical burden and after-hours work	Omissions, hallucinations, overreliance, narrative drift	Useful in adjacent clinical settings; only indirectly relevant to forensic reporting
On-premises or tightly sandboxed model with retrieval from approved sources	Best control over data flow and source base	Still needs expertise, maintenance, validation, and auditability	Often the most defensible architecture for institutions serious about forensic use
Specialized mental health note tool	Workflow integration and templating	Vendor claims may exceed evidence; security and ethics vary significantly	Only after careful procurement and validation
Device-regulated mental health AI product	Stronger formal oversight if it truly falls under device rules	Regulatory clearance does not by itself prove fitness for forensic report generation	Not a shortcut to forensic reliability

a LLM: large language model.

**Table 5.** Implementation steps, their necessity, and relevant practice implications.

Safe implementation step	Why it is necessary	How it should look in practice
Define the task boundary	Prevents slippage from clerical help into ghostwritten expert opinion	Written policy stating that interviews, formulation, reasoning, and ultimate opinions remain human-authored
Choose the environment before the model	Privacy and privilege risks arise from deployment context as much as model architecture	Approved secure platform, contract, retention policy, access controls, incident pathway
Validate locally	Vendor demonstrations do not test your population, workflows, or legal style	Pilot on historical or simulated forensic files with omission, hallucination, and bias scoring
Require source-linked outputs	Reduces ‘’bullshit’’ fluency by forcing traceability	Chronologies and summaries should point back to exact records, not free-floating prose
Mandate human verification and rewriting	Protects against omission and narrative drift	Reviewer checks every material statement against underlying documents and rewrites reasoning sections
Train clinicians and staff	Overreliance often reflects poor understanding rather than malice	Short mandatory training on limitations, confidentiality, prompting, and documentation
Keep an audit trail	Needed for accountability, disclosure, and learning from failure	Log tool, version, prompt template, inputs, reviewer, edits, final disposition
Define disclosure rules	Courts may require, or prudence may favor disclosure	Methodology section explains whether and how AI was used in bounded support tasks
Review performance over time	Models, products, and risks change	Quarterly audits of error patterns, complaints, near misses, and output drift

## AI vs Human Forensic Reasoning: The Appropriate Comparison

Importantly, the limitations raised as part of this viewpoint are not unique to AI. Human forensic experts are also susceptible to omission errors, confirmation bias, anchoring, allegiance effects, narrative framing, overconfidence, and disagreement in the interpretation of evidence. Cognitive biases have been documented across forensic mental health practice, and expert disagreement is an established feature of psycholegal evaluations even when experts review the same information. The appropriate comparison is, therefore, not between imperfect AI and an idealized human evaluator, but between 2 different systems of reasoning, each with distinct strengths, vulnerabilities, and mechanisms of error. The relevant question is not whether AI can make mistakes, since both humans and AI demonstrably do, but rather how AI-generated errors differ from human errors and what implications those differences have for forensic practice.

**Figure 2.**
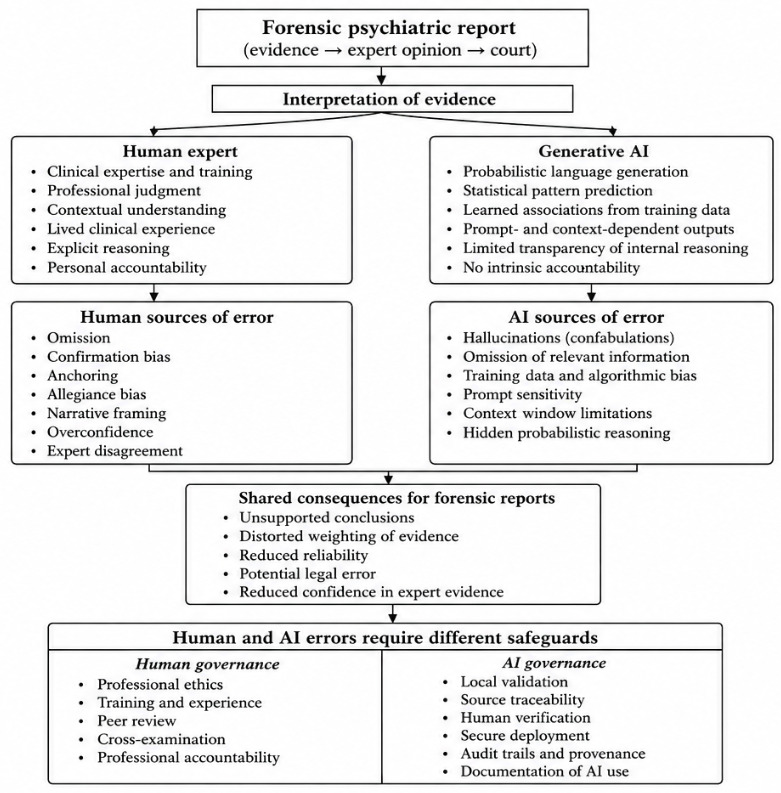
Distinct mechanisms of human and AI reasoning errors in forensic psychiatric report writing.

Human expert reasoning possesses characteristics that remain central to forensic testimony. Experts can explain how conclusions were reached, justify the relative weight assigned to competing pieces of evidence, acknowledge uncertainty, revise opinions in light of new information, and defend their reasoning during cross-examination. They also remain personally accountable for their opinions within professional, ethical, and legal frameworks. By contrast, contemporary LLMs generate probabilistic predictions based on learned statistical associations rather than transparent evidentiary reasoning. Their internal decision processes are not directly interpretable, the provenance of individual statements may be difficult to establish, and omissions or unsupported inferences may remain concealed within otherwise coherent narratives. These characteristics do not necessarily make AI less accurate than humans for every task, nor do they imply that human judgment is free from error. Rather, they suggest that AI requires a different governance framework, one emphasizing independent verification, source traceability, and explicit human accountability before its outputs can contribute to expert evidence. Distinct mechanisms of human and AI reasoning errors are presented in Figure 2.

## So... Can You Use AI for Forensic Psychiatry Report Writing?

In a strictly technical sense, yes. AI can assist with several components of the workflow, and the general medical documentation literature supports the idea that carefully supervised systems can reduce clerical burden and help process large volumes of text. Secure deployments may also exist that are contractually compatible with health data workflows. But the technically correct answer is yes only if “use” means constrained augmentation inside a validated, governed, source-checkable process. If “use” means asking a generator to write the expert report, formulate the psycholegal opinion, or transform confidential case materials in a public tool, the more accurate answer is no.

Should you use AI for forensic psychiatry report writing? You should probably use some form of AI eventually because the administrative and information management burden of forensic work is substantial, and the literature suggests that bounded automation can free up time and cognitive capacity for the parts of the job that most need human expertise. But you should use it conservatively, transparently, and in a way that protects the authenticity of the expert opinion. The ethically preferable model is “AI around the report” rather than “AI writes the report.” In other words, use it to prepare you to think, not to think for you.

A useful distinction is between clerical augmentation and epistemic reasoning. AI is well suited to bounded administrative tasks such as organizing documents, constructing chronologies, indexing records, transcribing interviews, or summarizing information already present in source materials. These activities are largely mechanical, reversible, and independently verifiable because the outputs can be systematically checked against the underlying documents. By contrast, psycholegal opinion formation is an interpretive process requiring the expert to weigh competing evidence, assess credibility, distinguish fact from inference, evaluate causation, apply legal standards, reconcile inconsistencies, and justify why one interpretation is more persuasive than another. These judgments are not merely clerical transformations of information but expert opinions that must remain transparent, accountable, and defensible under professional scrutiny and cross-examination. Consequently, the distinction is not that AI should be used ethically for some tasks but not others; rather, tasks that are readily verifiable are fundamentally different from those requiring accountable expert judgment.

Therefore, the cleanest practical position, especially from a scholarly viewpoint, is this. A forensic psychiatrist may appropriately use AI for secure preprocessing, administrative drafting support, and quality control assistance, provided that human review is rigorous, the reasoning and opinions are authored and adopted by the expert, material limitations are disclosed, and confidentiality and legal requirements are satisfied. A forensic psychiatrist should not use AI to generate or materially shape the substantive analysis or final opinion that the court is asked to trust as expert evidence. Based on the evidence available to date, there is insufficient evidence to support the use of generative AI for autonomous or materially influential psycholegal opinion generation.

## Open Questions and Limitations

The direct peer-reviewed literature on generative AI specifically for forensic psychiatry report writing remains limited. Most strong evidence comes from adjacent domains: clinical note generation, mental health LLM reviews, general expert witness AI guidance, and broader forensic bias scholarship. That means some recommendations here are principled inferences rather than conclusions from head-to-head forensic psychiatry trials. Those inferences are, however, in convergence with official guidance and in a defensible reading of what makes forensic reports epistemically and legally different from ordinary clinical writing.

Jurisdiction also matters. Disclosure rules, privilege doctrine, privacy obligations, and device regulation vary across countries and even within federated systems. The safest course in an unspecified jurisdiction is to assume that confidentiality, documentation, validation, and personal accountability will be scrutinized rather than forgiven. Until direct validation studies in forensic psychiatry become more numerous, conservative augmentation remains the strongest professional position.

Because AI evolves rapidly, conclusions regarding individual models should not be interpreted as permanent characteristics of future systems. The question is no longer whether AI should enter forensic psychiatry, but where its boundaries should be drawn. Current evidence supports augmentation of administrative work, not delegation of expert reasoning.

## Abbreviations

AAPL: American Academy of Psychiatry and the Law
COMPAS: Correctional Offender Management Profiling for Alternative Sanctions
HHS: US Department of Health and Human Services
HIPAA: Health Insurance Portability and Accountability Act
LLM: large language model
NIST: National Institute of Standards and Technology
OCR: Office for Civil Rights
